# Calcium imaging of primary canine sensory neurons: Small‐diameter neurons responsive to pruritogens and algogens

**DOI:** 10.1002/brb3.1428

**Published:** 2019-09-30

**Authors:** Joy Rachel C. Ganchingco, Tomoki Fukuyama, Jeffrey A. Yoder, Wolfgang Bäumer

**Affiliations:** ^1^ Department of Molecular Biomedical Sciences North Carolina State University College of Veterinary Medicine Raleigh NC USA; ^2^ Laboratory of Veterinary Pharmacology Azabu University Kanagawa Japan; ^3^ Institute of Pharmacology and Toxicology Faculty of Veterinary Medicine Freie Universität Berlin Berlin Germany

**Keywords:** dorsal laminectomy dissection, dorsal root ganglia cell culture, fura‐2AM, ratiometric calcium imaging

## Abstract

**Introduction:**

Rodent primary sensory neurons are commonly used for studying itch and pain neurophysiology, but translation from rodents to larger mammals and humans is not direct and requires further validation to make correlations.

**Methods:**

This study developed a primary canine sensory neuron culture from dorsal root ganglia (DRG) excised from cadaver dogs. Additionally, the canine DRG cell cultures developed were used for single‐cell ratiometric calcium imaging, with the activation of neurons to the following pruritogenic and algogenic substances: histamine, chloroquine, canine protease‐activated receptor 2 (PAR2) activating peptide (SLIGKT), compound 48/80, 5‐hydroxytryptamine receptor agonist (5‐HT), bovine adrenal medulla peptide (BAM8‐22), substance P, allyl isothiocyanate (AITC), and capsaicin.

**Results:**

This study demonstrates a simple dissection and rapid processing of DRG collected from canine cadavers used to create viable primary sensory neuron cultures to measure responses to pruritogens and algogens.

**Conclusion:**

Ratiometric calcium imaging demonstrated that small‐diameter canine sensory neurons can be activated by multiple stimuli, and a single neuron can react to both a pruritogenic stimulation and an algogenic stimulation.

## Graphical abstract

Establishing calcium imaging of dorsal root ganglia in dogs. Dogs are a translational model species but no functional data are available about DRG in dogs.

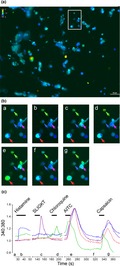

## 1 INTRODUCTION

The dorsal root ganglia (DRG) contain the cell bodies of afferent sensory nerves, and it is known that the same sensory receptors present at the neuromuscular junction or sensory terminals (postganglionic synapse) are also present on the cell bodies within the DRG (Davidson & Giesler, 2010; Malin, Davis, & Molliver, 2007). Activation of these sensory neurons by chemical substances can easily be evaluated by measuring the change in intracellular calcium in those cell bodies.

Collection and culturing of DRG from rodent species produced primary neuronal cultures that were used and are continuing to be used for understanding neurotransmission of itch and pain, even though translation and correlations to larger mammals have been questionable (Davidson et al., 2014; Mogil, 2009; Vierck, Hansson, & Yezierski, 2008). For example, it has been shown that pruritogenic substances activate mouse sensory neurons and that same substance injected intradermally in mice results in a significant scratch response (Akiyama et al., 2012; Schemann et al., 2012; Wilson et al., 2013), whereas scratching behavior was not replicated following intradermal injection of histamine, serotonin, tryptase, substance P, or interleukin‐2 in dogs (Carr, Torres, Koch, & Reiter, 2009), indicating there may be a species‐specific aspect to the neurotransmission of itch. Furthermore, several studies with substance P demonstrated clear activation of rodent sensory neurons and subsequent blockade modulating the pain response in mouse models (Pintér, Pozsgai, Hajna, Helyes, & Szolcsányi, 2014; Szallasi et al., 1999), but it is well known that the same promising results were not achieved in humans (Hill, 2000) suggesting that rodent models may be limited for its use for translational research to humans. Therefore, it is necessary to develop another animal model with DRG to elucidate a possible species difference in the neural activation by pruritogens and algogens. The dog is regarded as a valuable, translational model for atopic dermatitis and atopic itch, as it is a naturally occurring disease in dogs with increasing incidence (Marsella & De Benedetto, 2017). Dogs (as humans) respond with a characteristic pattern of lesions and (often severe) itch. However, our knowledge about itch transduction in dogs is poor, and to judge whether it is also translational in respect to itch (Martel, Lovato, Bäumer, & Olivry, 2017; Olivry & Bäumer, 2015), we wanted to study the canine DRG response to typical pruritogens described for humans and mice.

The interaction between itch and pain neurotransmission pathways is still under debate and continue to be studied. It is known that both itch and pain neurotransmission utilize small‐diameter neurons and ascend through the spinothalamic tract. The most commonly discussed theories are labeled‐line (specificity) theory, the intensity (pattern) theory, and the population coding theory (Akiyama & Carstens, 2013; Braz, Solorzano, Wang, & Basbaum, 2014; Davidson & Giesler, 2010; Duan et al., 2014; Handwerker & Schmelz, 2009; Meixiong & Dong, 2017; Mishra & Hoon, 2013; Schmelz, 2010; Zylka, Rice, & Anderson, 2005). The labeled‐line theory states there are specific afferent nerves that transmit only itch or only pain. While the intensity theory indicates that low‐intensity stimulations lead to an itch signal, increased intensity or the summation of stimulations lead to transmission of a pain signal. The population coding theory proposes that in addition to the labeled‐line theory there are crosstalk interactions (in the spinal cord or brain) controlling sensory transmission and although a nerve responds to a stimulus the subsequent perceived response may not correlate with that stimulus (e.g., an algogen may not induce a pain response). This study aims to incorporate both itch and pain transmission by exposure of sensory neurons to agonists that are determined to be either pruritogenic through histamine‐dependent pathways (histamine) and histamine‐independent pathways (chloroquine, bovine adrenal medulla peptide, canine protease‐activated receptor 2 activating peptide, compound 48/80, and 5‐hydroxytryptamine) or algogenic (substance P, allyl isothiocyanate, capsaicin) (Akiyama & Carstens, 2013; Akiyama, Carstens, & Carstens, 2010a; Davidson et al., 2014; Han et al., 2013; Metz, Grundmann, & Stander, 2011; Nakagawa & Hiura, 2013; Ohtori et al., 2002; Paus et al., 2006; Schemann et al., 2012; Valtcheva et al., 2016). The distinction of these algogenic substances only involved in pain has become less clear as current research has also shown their receptors associated with inducing itch and an off‐target activation of a pruriceptor by substance P (Akiyama et al., 2013; Azimi et al., 2017; Feng et al., 2017; Fukuyama, Ganchingco, Mishra, et al., 2017b; Ständer & Yosipovitch, 2019). Receptors for these agonists are mainly G protein‐coupled receptors, including tachykinin receptor‐1, histamine‐1 receptor, sensory neuron receptor, protease‐activated receptor 2, and mas‐related G protein‐coupled receptors (Mrgprs). While the transient receptor potential cation channel subfamily V member 1 (TRPV1) and subfamily A member 1 (TRPA1) are ion channels, chemical ligand binding to each of these receptors leads to neuronal activation marked by an increase in intracellular calcium.

Previous studies with canine DRG describe the presence of small (<40 µm)‐ and large‐diameter (>40 µm) neurons with the characteristic in situ and culture appearance as described in rodent and human DRG (Gerhauser, Hahn, Baumgärtner, & Wewetzer, 2012; Rosati et al., 2012; Tongtako et al., 2017). Additionally, canine DRG were shown to express substance P‐associated tachykinin receptor 1 (TACR1) and the voltage‐gated calcium channel subunit, TRPV1, and histamine receptors (H_1,2,3,4_R) (Rosati et al., 2012; Rossbach & Bäumer, 2014). Similar to adult rodent studies, adult canine DRG culturing is simple as it does not need additional supplementation in culture to remain viable and initiate neurite growth (Gerhauser et al., 2012; Malin et al., 2007).

The purpose of this study was to provide a simple, rapid dissection procedure for canine DRG extraction from cadavers, a repeatable method of processing those canine DRG for cell culture, and single‐cell ratiometric calcium imaging of canine sensory neurons. Additionally, this study will demonstrate the functional validation of previous molecularly described receptors on canine DRG and biological activity of chemical stimulations previously used in human and rodent calcium imaging studies of sensory neurons.

## 2 MATERIALS AND METHODS

### 2.1 Materials

All reagents and materials were obtained from Sigma‐Aldrich unless otherwise stated. DRG cell culture media was composed of Dulbecco's Modified Eagle Medium (DMEM; Corning), 10% fetal bovine serum (FBS; Atlanta Biologicals, Flowery Branch), and 1% penicillin streptomycin (Pen Strep; Corning). All equipment for calcium imaging was obtained from Warner Instruments.

### 2.2 Dissection and cell culture preparation

Prior to dissection, autoclaved glass coverslips (#1.5 18 mm; Warner Instruments) were placed into a 12‐well plate with 25 μl of DMEM with poly‐L‐lysine (0.095 mg/ml) and laminin (5 mg/ml; Corning) added just to the center of slide to dry overnight and then gently washed with sterile water, allowed to air dry and stored in horizontal laminar flow hood before use.

**Figure 1:**
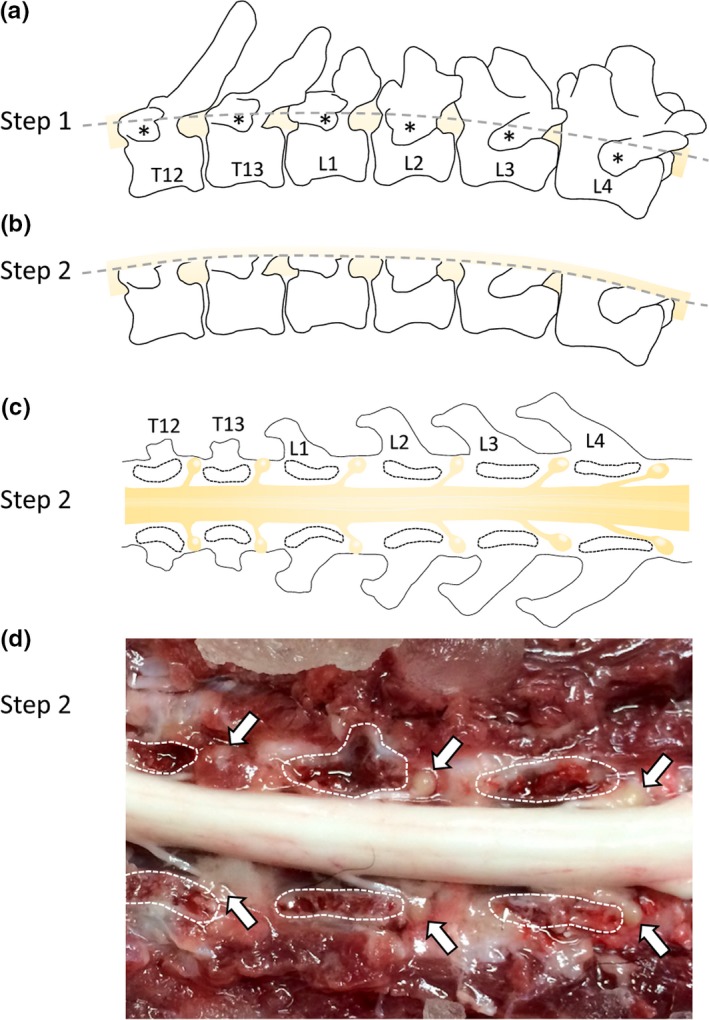
Schematic diagram for dorsal laminectomy dissection of canine dorsal root ganglia. (a) Lateral view of canine thoracolumbar vertebral column and spinal cord position, (*) denotes the transverse processes as the dorsal landmarks for accessing the underlying pedicles to transect for (b) exposing the spinal cord. (c) Dorsal view of canine thoracolumbar vertebral column with the cut surface of pedicle outlined. (d) Dorsal view following dissection, with the cut surface of pedicle outlined for landmark orientation and white arrows pointing at in situ DRG. Thoracic (T) and lumbar (L) vertebrae labeled for referencing

Samples were obtained from a total of 15 adult dogs euthanized for reasons unrelated to this study. Dogs were euthanized at other facilities following American Veterinary Medical Association (AVMA) guidelines and in accordance with North Carolina state law using FDA‐approved euthanasia solution at labeled doses (390 mg/ml pentobarbital sodium and 50 mg/ml phenytoin sodium). Cadavers were transported to North Carolina State University necropsy for dissection. Cadavers were placed in sternal recumbency, and twelve DRG from the thoracic‐lumbar region (T6‐L6) were collected through a dorsal approach by removing epaxial muscles, dorsal spinous processes, and removal of the dorsal portion of the spinal canal by cutting through the pedicles located just ventral to the transverse processes (Figure 1). This dissection method allowed for dorsolateral retraction of the spinal cord and visualization of the DRG for excision using the visible cut surface of the pedicle and thoracolumbar junction (T13‐L1) as landmarks. As with other mammals, the dorsal root ganglion can be identified by following the caudally projecting dorsal spinal root to the distal aspect before it passes through the intervertebral foramen, pictured in Figure 1. The DRG excised were transported in PBS containing 1% penicillin streptomycin on ice to the laboratory for immediate processing. Several cuts through the meningeal layer surrounding each DRG was made before placement in an untreated 35‐mm tissue culture Petri dish and enzymatically softened with 2 ml of collagenase XI (10 mg/ml) in dispase (5 U/ml; STEMCELL Technologies) for 30 min at 37°C and 5% CO_2_. Continued dissociation was done by graduated mechanical trituration with fire‐polished Pasteur pipettes. Large cells and nondiscriminatory adhering cells (i.e., fibroblasts) were allowed to settle and adhere to the Petri dish during an additional 30 min at 37°C and 5% CO_2_. The cell suspension was collected and washed with cell culture media by centrifugation at 290 g for 5 min. The cell suspension pellet was resuspended in 160 μl media, and 20 μl of cell suspension was seeded onto pretreated glass coverslips, with surrounding unused wells filled with sterile water to increase plate humidity and prevent desiccation of cell suspension during targeted adherence at 37°C and 5% CO_2_ for 3 hr. The nonadherent cells (i.e., nonviable neurons) were gently washed away by slowly flooding wells with fresh media and then returned to incubate overnight.

### 2.3 Immunofluorescence

Within 24 hr from dissection, the slides with cultured cells were acetone‐fixed and stored at −20°C until immunocytochemical analysis. Cells were incubated with primary antibody anti‐Neu‐N (Aves Lab), which specifically binds the nucleus of neurons, secondary antibody goat anti‐chicken IgG (Santa Cruz Biotechnology), and mounted with UltraCruz with DAPI mounting media (Santa Cruz Biotechnology). All immunofluorescence images were taken on Leica DM5000B with Leica Application Suite X (1.5.1.13187) software.

### 2.4 Calcium imaging

The single‐cell intracellular calcium measurements with fura‐2‐acetoxymethyl ester (Fura‐2 AM; Invitrogen) were performed with modifications to a previous publication by our group (Fukuyama, Ganchingco, & Bäumer, 2017a). Imaging of the heterogeneous primary canine sensory neuron culture was performed early (between 18 and 24 hr postdissection) to ensure single‐cell identification and avoid interference from neural networking (Malin et al., 2007). The glass coverslips were fitted into the corresponding QR chamber + QE‐1 platform (Warner Instruments) on a Nikon TE200 microscope. The temperature of platform and in‐line solution was controlled to 34 ± 2°C with the dual channel heater controller (Warner Instruments), and the bathing solution (pH 7.4, 136 mM NaCl, 5.6 mM KCl, 1.2 mM MgCl_2_, 2.2 mM CaCl_2_, 1.2 mM NaH_2_PO_4_, 14.3 mM NaHCO_3_, and 10 mM D‐glucose) was continuously flowed through the perfusion chamber at 3 ml/min. To avoid direct application and temperature change artifacts, the following compounds were delivered in‐line: histamine (100 µmol/L), chloroquine (10 µmol/L), canine protease‐activated receptor 2 activating peptide (SLIGKT, 500 µmol/L; Thermo Scientific Pierce), compound 48/80 (1 mmol/L), 5‐hydroxytryptamine receptor agonist (5‐HT, 1 mmol/L), bovine adrenal medulla peptide (BAM8‐22, 1 µmol/L), substance P (100 µmol/L), allyl isothiocyanate (AITC, 10 nmol/L), and capsaicin (1 µmol/L). Only one field of view was imaged per slide with no more than five chemical substances applied, and at least, 1‐min interval between chemical applications was allowed for return to a steady resting baseline 340:380 level. The concentrations selected were based on unpublished pilot experiments our group performed with canine DRG and previous studies done with human and murine DRG (Akiyama, Carstens, & Carstens, 2010b; Akiyama et al., 2012; Azimi et al., 2017; Davidson et al., 2014; Fukuyama, Ganchingco, & Bäumer, 2017a; Fukuyama, Ganchingco, Mishra, et al., 2017b; Fukuyama et al., 2018; Liu et al., 2016; Schemann et al., 2012; Valtcheva et al., 2016; Wilson et al., 2011, 2013). Ratiometric Fura‐2 AM UV imaging was performed with continuous capture and automatic Lambda optical filter changer alternating fluorescence excitation at 340 and 380 nm every 100 ms (emission at 510 nm), with the NIS‐Elements imaging software (Nikon Instruments) calculating the 340:380 ratio values and assigning the color spectral designation to the value. Following chemical addition, an increase in 340:380 directly reflected an increase in Fura‐2 detecting intracellular calcium and therefore activation of the sensory neuron. Neurons were noted to have a positive response (i.e., responsive) if the 340:380 ratio value increased by more than 10% of the resting baseline 340:380 level prior to chemical application (Akiyama et al., 2010b; Fukuyama, Ganchingco, & Bäumer, 2017a). The total number of neurons analyzed per dog and the percent of neurons responsive are provided in Table 1.

**Table 1:** Summary of neurons analyzed and responsive per dog

Dog	Number of slides analyzed per dog	Total number of neurons analyzed	% neurons responsive^a^
1	7	413	70.0
2	6	435	79.3
3	8	391	70.3
4	8	471	51.4
5	8	622	66.1
6	5	331	54.1
7	7	273	34.4
8	6	339	46.9
9	5	337	87.8
10	4	232	38.4
11	4	253	74.7
12	7	246	50.4
13	6	206	66.5
14	3	173	83.8
15	3	123	84.6

a Percent of analyzed neurons responsive (with a positive response determined to be at least >10% increase in 340:380 from baseline 340:380 level) to at least one chemical substance.

### 2.5 Statistical analysis

Area‐proportional Venn diagrams were constructed using euler*APE* published by Micallef and Rodgers (2014).

## 3 RESULTS

### 3.1 Primary sensory neuron culture from canine dorsal root ganglia

**Figure 2:**
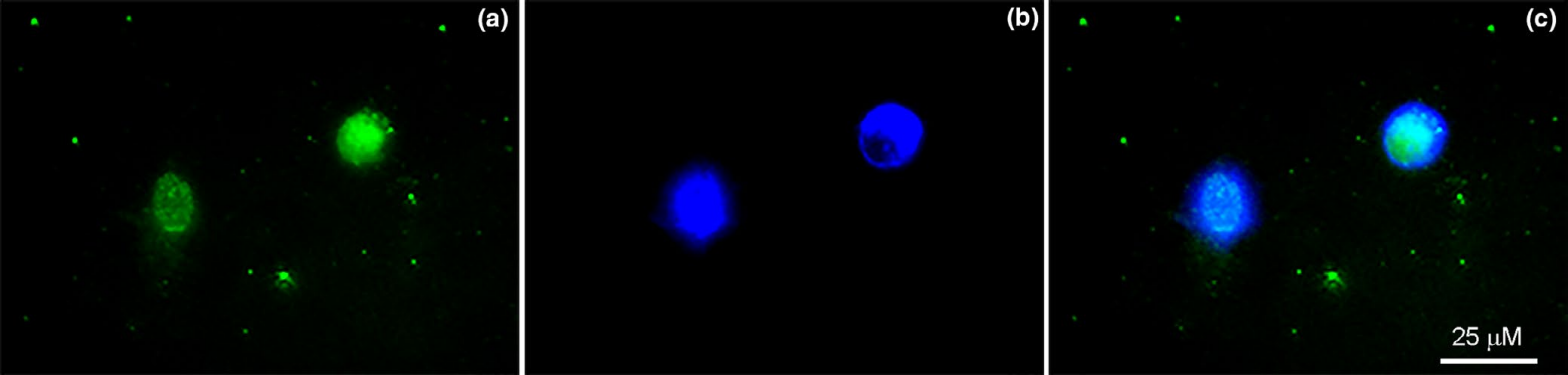
Verification of adherent sensory neurons in culture following processing of canine dorsal root ganglia. Immunolabeling of the heterogeneous adherent canine DRG cell culture demonstrating (a) NeuN (known to be specific for neurons) with (b) DAPI colocalizing in the nucleus of sensory neurons, and a (c) merged image conveying the heterogeneous nature of the culture and identifying neurons. 40× magnification

This study achieved a simple dissection approach for isolation of canine DRG which decreased the transition time of processing DRG tissue for culture. Additionally, the optimized rapid mechanical and enzymatic dissociation repeatedly produced viable primary canine neuron cultures. The cultured primary canine neurons cultured displayed co‐localization of NeuN with DAPI (Figure 2). Dissected canine DRG tissue were successfully dissociated into heterogeneous single‐cell cultures predominantly small‐diameter neurons (i.e., sensory neurons), large‐diameter neurons, and satellite cells (Figure S1) similar to previously published canine DRG studies (Gerhauser et al., 2012; Rosati et al., 2012; Tongtako et al., 2017). The small‐diameter sensory neurons analyzed within this study ranged from 8 to 28 µm in diameter (18.9 µm median cell size, data not shown).

**Figure 3:**
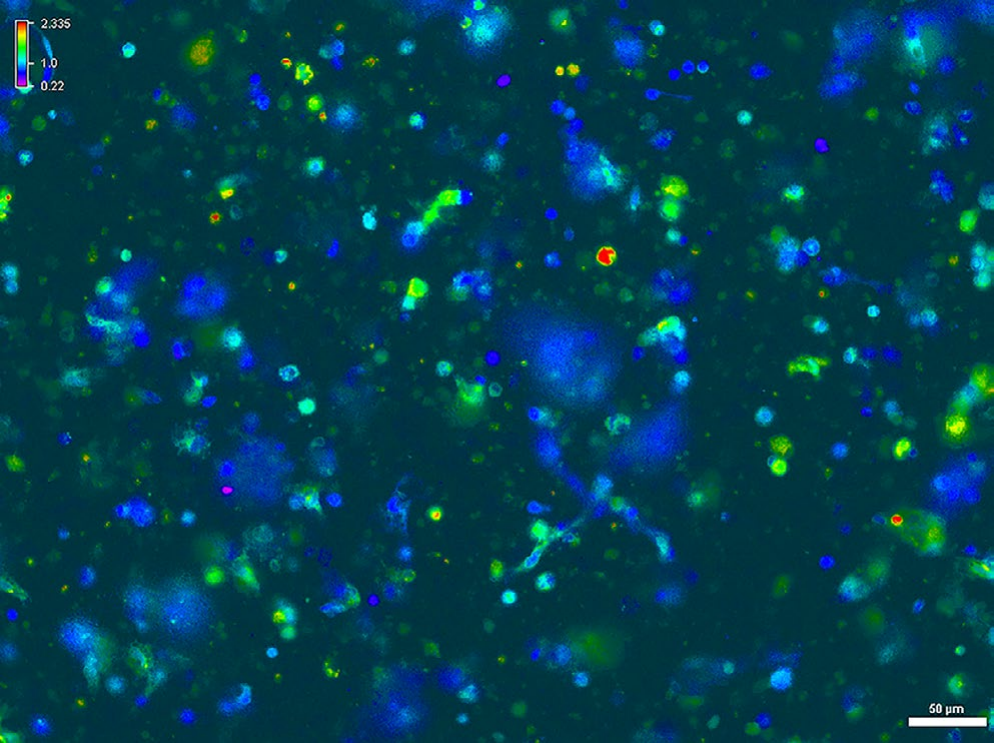
Representative image capture demonstrating the intracellular fluorescence variation of canine sensory neurons at baseline (unstimulated) levels with Fura‐2 a.m. incubation. The variation in the basal fluorescence highlights the importance of the Nikon Elements software continuously calculating and displaying the 340:380 ratio. Software assigns color within spectrum (top left) based on the calculated 340:380 value. 200× magnification

Based on morphological appearance at 200× magnification, neurons selected for analysis were 10–30 μm in diameter with a distinct nucleus. Viable sensory neurons from the canine DRG cell cultures remained adherent to the glass slide following cytoplasmic incorporation of Fura‐2 AM and throughout imaging. Figure 3 depicts a representative single‐frame capture of the 340:380 ratiometric image with the color spectral display of the heterogeneous primary canine sensory neuron culture within 24 hr of dissection. This single‐frame capture shows the variation in starting intracellular calcium levels, which enabled exclusion of neurons with starting 340:380 baseline levels 1.2 or greater.

### 3.2 Activation of canine sensory neurons following pruritogenic and algogenic exposures

**Figure 4:**
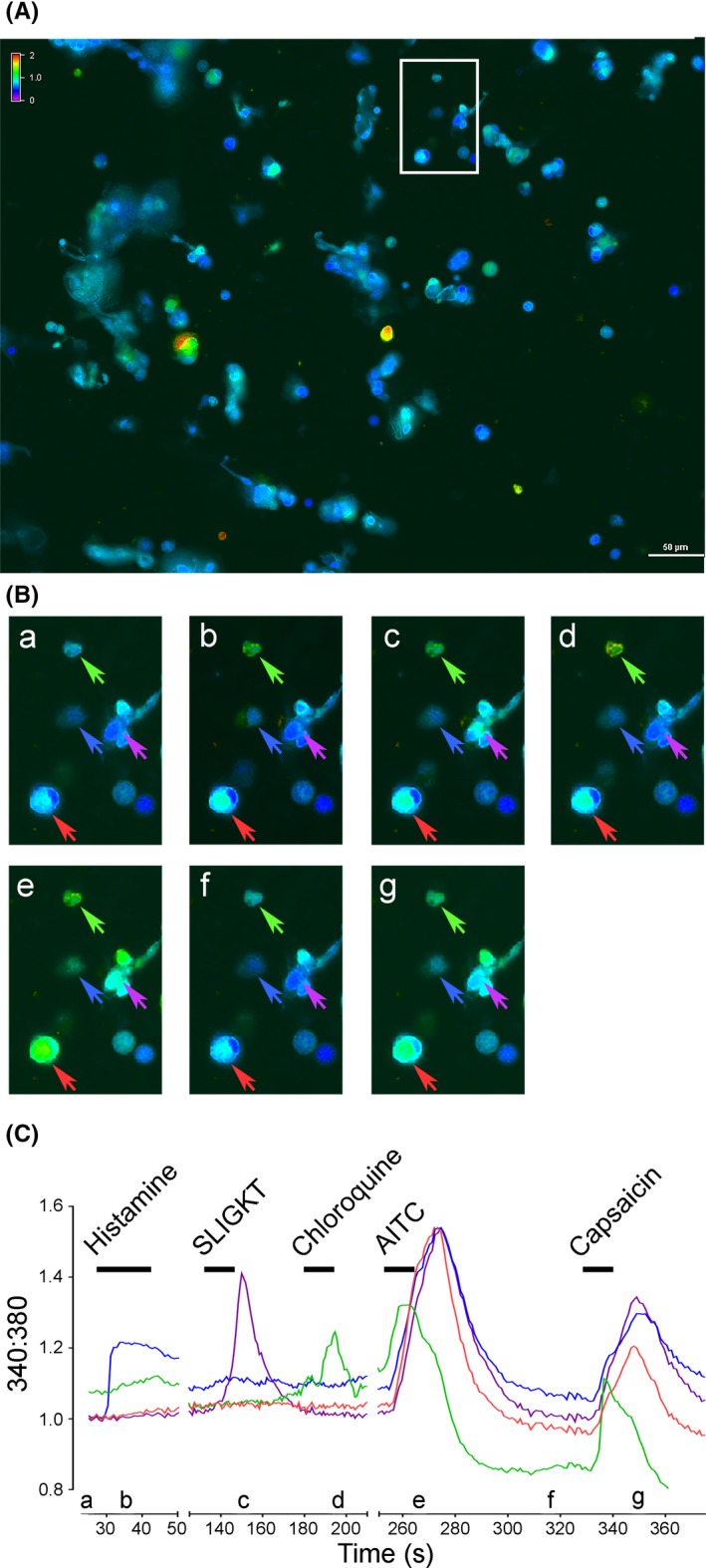
Multiple canine sensory neurons show reactivity to more than one chemical exposure. (A and B) Representative single‐frame capture images demonstrating visual change in color spectral display of multiple canine DRG neurons associated with the change in 340:380 levels. The increased 340:380 directly correlates to a transient increase in intracellular calcium (demonstrating neuronal activation) following chemical exposures with histamine, SLIGKT (canine PAR2 agonist), chloroquine, allyl isothiocyanate (AITC), and capsaicin. White rectangle in A is region imaged in B. Each image in B (a–g) corresponds to a single‐frame capture in the sequence taken at the time denoted by its location along the time axis in C. Colored arrows correspond to the neurons in each image of B (a–g) associated with the red, blue, green, and purple colored traces of the change in 340:380 over time (s) shown in C. Bars in C denotes duration of chemical exposures

A total of 4,992 neurons were analyzed for responsiveness to the standard stimulants histamine and capsaicin. Additional exposures to other chemical substances were also performed, where 1,201 of the total 4,992 neurons were also exposed to 5‐HT; 1,996 to SLIGKT; 2,047 to chloroquine; 1,454 to BAM8‐22; 1,819 to compound 48/80; 1,454 to substance P; and 1,431 to AITC. All chemical exposures started with histamine followed by randomized addition of a maximum of three other chemical substances before exposure with capsaicin. No order effects were observed with the randomized chemical additions. Each chemical substance elicited a positive responsive within 30 s from application. Multiple canine sensory neurons showed activation following series of chemical exposures marked by an increase in 340:380, an example color spectral display of activated neurons and corresponding trace of 340:380 levels shown in Figure 4.

Table 2 summarizes the number of neurons responsive to each chemical substance. Of the 64.86% of neurons noted to be active (3,238 active neurons/4,992 neurons analyzed), 64.89% (2,101 responsive neurons/3,238 active) were shown to only be responsive to one chemical substance, 34.28% (1,110/3,238) were responsive to two chemical substances, and the remaining 0.83% (27/3,238) responsive to three or four chemical substances. No single neuron analyzed was shown to be responsive to all five chemical exposures.

**Table 2:** Summary of canine sensory neurons responsive to each chemical stimulation^a^

Totals	H	5‐HT	SLIGKT	C	C48/80	BAM8‐22	Sub P	AITC	Cap
Number of responsive	392	62	46	34	177	23	27	887	2,774
Number of active^b^	3,238	714	1,346	1,369	1,046	903	903	1,095	3,238
% responsive of active^c^	12.11	8.68	3.42	2.48	16.92	2.55	2.99	81.00	85.67

a Histamine (H), serotonin agonist (5‐HT), canine protease‐activated receptor 2 (PAR2) agonist (SLIGKT), chloroquine (C), compound 48/80 (C48/80), bovine adrenal medulla peptide (BAM8‐22), substance P (Sub P), allyl isothiocyanate (AITC), capsaicin (Cap).

b The number of viable active neurons exposed to each chemical stimulation.

c The percentage of active neurons shown to be responsive (with a positive response determined to be at least >10% increase in 340:380 from baseline 340:380 level) to each chemical stimulation.

**Figure 5:**
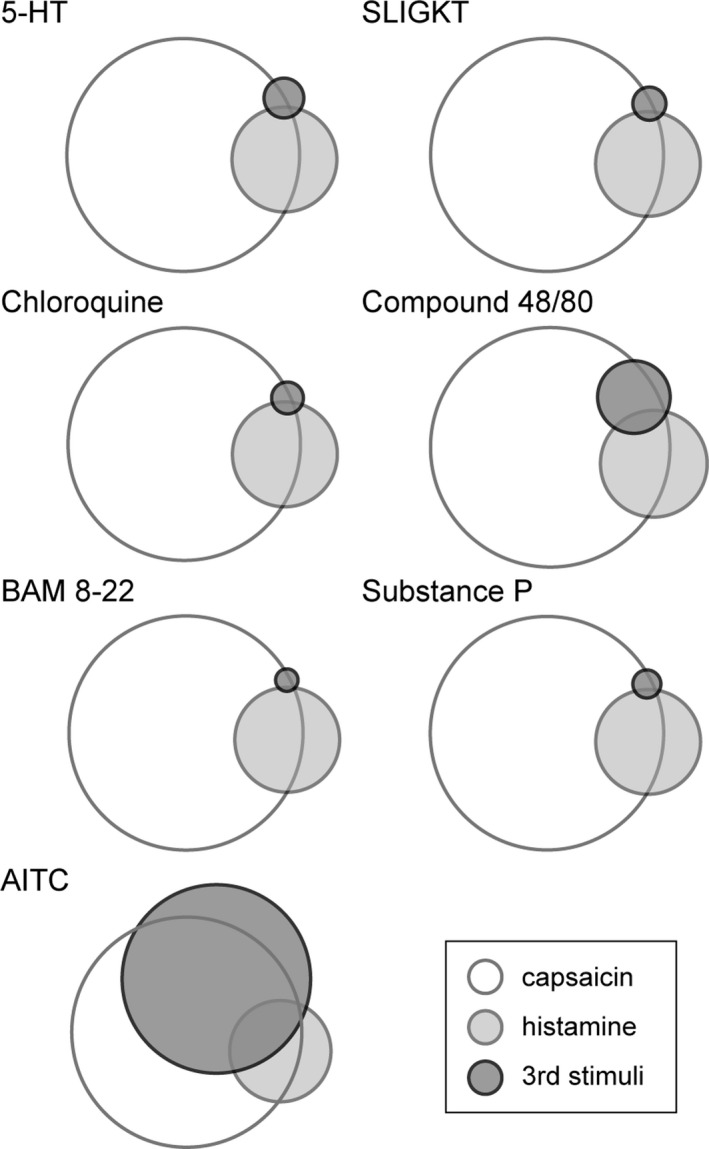
Canine sensory neurons can show specific reactivity to only one chemical exposure or to multiple chemical exposures, demonstrating multiple pathways of pruritogenic or algogenic activation can be through a single neuron. Area‐proportional Venn diagrams reflect the relative proportions of canine sensory neurons responsive to stimulation by capsaicin (white), histamine (light gray), and another chemical stimulant (dark gray). Of the histamine responsive neurons, 41.6% were only responsive to histamine and 57.9% were concurrently responsive to capsaicin. Of the capsaicin responsive neurons, 70.0% were only responsive to capsaicin. Other relative proportion values are listed in Table S1

All neurons analyzed in this study were exposed to the known pruritogen histamine and the known algogen capsaicin. The percent of neurons responsive to capsaicin was 85.67% (2,774 responsive neurons/3,238 active neurons) in comparison with 12.11% (392/3,238) of neurons responsive to histamine, with 7.01% of all neurons responsive to both (227/3,238). The area‐proportional Venn diagrams depict the relative proportional relationship of neurons responsive to a third chemical substance in relation to the pruritogenic (histamine responsive) and algogenic (capsaicin responsive) neurons, highlighting the relationship of the neuronal response to pruritogen versus algogen (Figure 5). The proportion of neurons activated by a chemical substance other than capsaicin and histamine showed a significant percentage of those neurons that were concurrently responsive to capsaicin compared with a low proportion of those neurons also responsive to histamine (Figure 5, percentages listed in Table S1).

## 4 DISCUSSION

This is, to our knowledge, the first publication using canine DRG culture for single‐cell ratiometric Fura‐2 AM UV imaging of transient intracellular calcium to demonstrate neuronal activation. This study demonstrates successful development of a simple primary canine DRG neuronal culture from canine cadavers. Utilizing dogs euthanized for reasons unrelated to this study was advantageous for tissue collection but posed difficulties that had to be overcome by this presented study. With the delayed time from euthanasia and transportation to dissection, all cadavers were covered in ice during dissection to increase viability. The optimal time from euthanasia to completing DRG excision for processing was determined to be 2 hr or less, and the optimal amount of time for processing the DRG tissue for culture was determined to be 1 hr or less. Positioning in canine cadavers is easier in sternal recumbency to access the spinal column due to their quadruped anatomy compared with dorsal recumbency as done in human cadavers (Valtcheva et al., 2016). Although the dorsal laminectomy approach was determined to be a less rapid method for isolation in rodents (Sleigh, Weir, & Schiavo, 2016), this approach is most efficient in dogs due to their larger size, increased musculature, and the increased size and density of their skeleton. Additionally, the rapid processing of DRG tissue developed in this study increased neuronal cell viability and improved reproducible cultures of predominantly sensory neurons (i.e., small‐diameter neurons).

Dynamic calcium imaging of the primary canine sensory neuron culture confirmed biological activity of chemical substances on canine neurons and a viable neuronal population with functional receptors. Chemical substances chosen for this study were based on known pruritogens and algogens with a focus on specific agonists for receptors known to be expressed on canine DRG (Rossbach & Bäumer, 2014) and agonists used in previous murine calcium imaging studies (Akiyama et al., 2010b, 2012; Fukuyama, Ganchingco, & Bäumer, 2017a; Fukuyama, Ganchingco, Mishra, et al., 2017b; Fukuyama et al., 2018; Liu et al., 2016; Schemann et al., 2012; Wilson et al., 2011, 2013). In comparison with previously published data of ratiometric calcium imaging in rodent studies, there is a similar proportion of canine neurons shown to be responsive to histamine (6%–25% in rodent studies), 5‐HT (4%–9%), and compound 48/80 (9%–29%). There are less canine neurons responsive to chloroquine (6%–15%) and BAM8‐22 (3%–7%). There are more canine neurons responsive to capsaicin (45%–75%) and significantly more responsive to AITC (28%–38%). While SLIGKT peptide is the canine‐specific PAR2 agonist, the proportion of responsive canine sensory neurons is comparable to the proportion of murine neurons responsive to the murine‐specific SLIGRL (2%–12%). (Akiyama et al., 2010b, 2012; Fukuyama, Ganchingco, & Bäumer, 2017a; Fukuyama, Ganchingco, Mishra, et al., 2017b; Fukuyama et al., 2018; Liu et al., 2016; Schemann et al., 2012; Wilson et al., 2011, 2013). This difference in neuronal activation shows a possible species‐specific activation of neurons and warrants further investigation into causation. Based on previously published data by our group, we know that in vivo exposures can affect sensory neuron activity (Fukuyama, Ganchingco, & Bäumer, 2017a; Fukuyama et al., 2018) and therefore cannot rule out that the unknown history of the canine cadavers contributes to these differences. We accounted for this possible variability by increasing the data collected from altogether 15 dogs and over 4,000 individual neurons. Utilizing canine DRG from unknown cadavers in neurotransmission studies is more advantageous than rodent models for translation into clinical studies as it incorporates the confounding variables of an extended lifespan, environmental exposures, possible exposure to diseases and vaccinations, and development of naturally occurring disease conditions.

There was a population of neurons that were responsive to only histamine, only nonhistamine pruritogens, and neurons responsive to both. This confirms the idea of separate histamine‐dependent and histamine‐independent itch neurotransmission pathways and suggests the hypothesis that a subset of small‐diameter neurons which will nonpreferentially transmit itch. However, confirming neuronal activation by histamine, 5‐HT, and substance P further confounds the lack of scratching behavior elicited in dogs by these chemical substances in a previous study by Carr et al. (2009). We can conclude that canine neurons are responsive to these pruritogens and postulate, as supported by the population coding theory, that transmission of itch to elicit a scratch response in dogs is dependent on subsequent crosstalk interactions or interneurons within the spinal cord or brain.

Based on the experimental design of this presented study, all sets of neurons analyzed were exposed to both histamine and capsaicin to demonstrate the relationship of pruritogenic and algogenic activation on canine sensory neurons. There were sensory neurons exhibiting only functional pruriceptors by their sole response to pruritogens and sensory neurons exhibiting only nociceptors by their sole response to algogens, indicative of the labeled‐line theory. However, similar to previous studies there is a significant proportion of neurons responsive to a pruritogen and capsaicin concluding concurrent expression of pruriceptors and TRPV1 on these peripheral sensory neurons, demonstrating there are not separate pain and itch sensory neurons (Akiyama et al., 2010a, 2012; Akiyama, Merrill, Carstens, & Carstens, 2009; Akiyama, Tominaga, Takamori, Carstens, & Carstens, 2014; Gold, Dastmalchi, & Levine, 1996; Lu, Zhang, & Gold, 2006; Nakagawa & Hiura, 2013). Current research suggests that TRPV1 is involved in itch, and therefore, activation of these neurons may still confer itch transmission (Feng et al., 2017; Fukuyama, Ganchingco, Mishra, et al., 2017b). Additionally, TRPA1 was shown to be necessary for itch transmission (Feng et al., 2017). Furthermore, substance P was shown to be biologically active on a pruriceptor and the NK‐1 receptor involved in itch transmission (Akiyama et al., 2013, 2015; Azimi et al., 2017). The distinction of pain transmission by algogens and nociceptors continues to overlap with itch transmission, building evidence for the population coding theory. Unfortunately, this in vitro study cannot make conclusions about the intensity theory, as analysis only demonstrates neuronal activation.

## 5 CONCLUSION

Based on these results, the interaction between itch and pain neural processing is not as clear as one theory; it exemplifies neural plasticity, neural selectivity likely based on previous exposures leading to sensitization, and the idea of multimodal peripheral neurons which then rely on interneurons within the dorsal horn of the spinal cord or other central processing for transmission of the itch or pain signal.

This presented study demonstrates that single‐cell calcium imaging can reliably be accomplished in a nonrodent model through a simple dissection‐culturing method and confirmed functional receptors present on canine sensory neurons with biologically active pruritogens and algogens. This method can be used for further studies in altering peripheral neurotransmission, understanding peripheral sensitization, testing new ligands for activation or inhibition of pain and itch sensation, and can be used for studying naturally occurring or chronic conditions in a large mammal model.

## CONFLICT OF INTEREST

The authors have no conflict of interests to state.

## Data Availability

The data that support the findings of this study are available from the corresponding author upon reasonable request.
