# Supplementary material for: Calcium imaging of primary canine sensory neurons: Small‐diameter neurons responsive to pruritogens and algogens
Source: Brain Behav. 2019 Sep 30;9(12):e01428. doi: 10.1002/brb3.1428 (PMC6908857; doi:10.1002/brb3.1428)
Supplement: Supplementary file 1 [file BRB3-9-e01428-s001.pdf]

Calcium Imaging of Primary Canine Sensory Neurons: Small Diameter Neurons Responsive to Pruritogens and Algogens

Joy Rachel C. Ganchingco, Tomoki Fukuyama, Jeffrey A. Yoder, and Wolfgang Bäumer

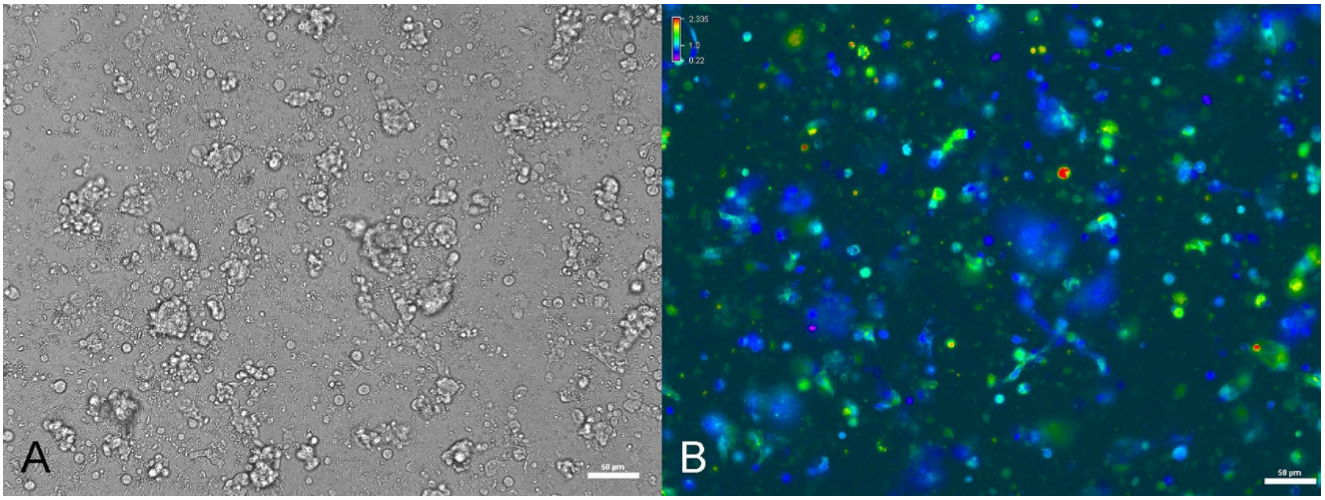

Supplemental Figure 1. Primary canine sensory neuron culture. (A) Brightfield image of heterogeneous primary culture includes large and small diameter neurons. **(B)** Image capture demonstrating the variation in the background and baseline fluorescence of cells shown in (A) following incubation with Fura2-AM (shown in Figure 3). Software assigns color within spectrum (top left) based on calculated 340:380 value. 200x magnification. Bar = 50 μM.
