# Supplementary material for: Calcium imaging of primary canine sensory neurons: Small‐diameter neurons responsive to pruritogens and algogens
Source: Brain Behav. 2019 Sep 30;9(12):e01428. doi: 10.1002/brb3.1428 (PMC6908857; doi:10.1002/brb3.1428)
Supplement: Supplementary file 2 [file BRB3-9-e01428-s002.pdf]

# Calcium Imaging of Primary Canine Sensory Neurons: Small Diameter Neurons Responsive to Pruritogens and Algogens

Joy Rachel C. Ganchingco, Tomoki Fukuyama, Jeffrey A. Yoder, and Wolfgang Bäumer

**Supplemental Table 1. Percent overlap of responsive neurons to individual stimuli and histamine and/or capsaicin[1]**

| | Histamine | Capsaicin | Histamine & Capsaicin | Neither[2] |
|---|---|---|---|---|
| 5-HT | **6.5** | **43.5** | **6.5** | **43.5** |
| SLIGKT | **4.3** | **41.3** | **6.5** | **47.8** |
| Chloroquine | **11.8** | **50.0** | **8.8** | **29.4** |
| Compound 48/80 | **0.6** | **84.2** | **5.6** | **9.6** |
| BAM 8-22 | **0.0** | **43.5** | **8.7** | **47.8** |
| Substance P | **3.7** | **66.7** | **3.7** | **25.9** |
| AITC | **0.8** | **70.5** | **5.9** | **22.9** |

[1] As shown in Figure 5.
[2] Percent of neurons not responsive to histamine or capsaicin, but still responsive to that individual chemical stimulant.
